# Test–retest reliability of the Epworth Sleepiness Scale in clinical trial settings

**DOI:** 10.1111/jsr.13476

**Published:** 2021-09-20

**Authors:** Russell Rosenberg, Kimberly Babson, Diane Menno, Susan Morris, Michelle Baladi, Danielle Hyman, Jed Black

**Affiliations:** ^1^ NeuroTrials Research Atlanta GA USA; ^2^ Atlanta School of Sleep Medicine Atlanta GA USA; ^3^ Jazz Pharmaceuticals Palo Alto CA USA; ^4^ Stanford Center for Sleep Sciences and Medicine Palo Alto CA USA

**Keywords:** JZP‐110, repeated measurements, reproducibility, Sunosi, surveys and questionnaire, wake‐promoting agents

## Abstract

The present analysis examined the test–retest reliability of the Epworth Sleepiness Scale in participants with excessive daytime sleepiness associated with narcolepsy or obstructive sleep apnea in three clinical trials. Intraclass correlation coefficient estimates for Epworth Sleepiness Scale scores from two solriamfetol 12‐week placebo‐controlled trials (one narcolepsy, one obstructive sleep apnea) and one long‐term open‐label extension trial (narcolepsy or obstructive sleep apnea) were calculated using postbaseline time‐point pairs for the overall population in each trial, by treatment, and by primary obstructive sleep apnea therapy adherence. In the 12‐week narcolepsy trial, intraclass correlation coefficients (95% confidence intervals) were 0.83 (0.79, 0.87) for weeks 4 and 8 (*n* = 199), 0.87 (0.83, 0.90) for weeks 8 and 12 (*n* = 196), and 0.81 (0.76, 0.85) for weeks 4 and 12 (*n* = 196). In the 12‐week obstructive sleep apnea trial, intraclass correlation coefficients (95% confidence intervals) were 0.74 (0.69, 0.78) (*n* = 416), 0.80 (0.76, 0.83) (*n* = 405), and 0.74 (0.69, 0.78) (*n* = 405), respectively. In the open‐label extension trial, intraclass correlation coefficients (95% confidence intervals) were 0.82 (0.79, 0.85) for weeks 14 and 26/27 (*n* = 495), 0.85 (0.82, 0.87) for weeks 26/27 and 39/40 (*n* = 463), and 0.78 (0.74, 0.81) for weeks 14 and 39/40 (*n* = 463). Placebo/solriamfetol treatment or adherence to primary obstructive sleep apnea therapy did not affect reliability. In conclusion, across three large clinical trials of participants with narcolepsy or obstructive sleep apnea, Epworth Sleepiness Scale scores demonstrated a robust acceptable level of test–retest reliability in evaluating treatment response over time.

## 1 INTRODUCTION

The Epworth Sleepiness Scale (ESS) is a patient‐reported questionnaire that measures excessive daytime sleepiness (EDS) by assessing situational sleep propensities (Johns, 1991). Specifically, the ESS is composed of eight items that assess the likelihood of falling asleep in real‐world situations, such as reading, watching television, or driving. Each item is scored from zero to 3, for a total score of zero to 24, with higher scores indicating a greater severity of EDS. Scores of ≤10 are commonly considered within the normal range, whereas scores of >10 indicate pathological EDS (Johns & Hocking, 1997; Johns, 1991). Data from patients with obstructive sleep apnea (OSA) suggest that changes of 2–3 points may be considered the minimum clinically important difference on the ESS (Crook et al., 2019; Patel et al., 2018).

The ESS is widely used in sleep research, clinical trials, and clinical practice. In clinical trials, the ESS is often used to evaluate the effects of treatment intervention on EDS in several disease states, including narcolepsy and OSA. For instance, the efficacy and regulatory approvals of several wake‐promoting agents, such as solriamfetol, modafinil, armodafinil, and pitolisant, has been supported by reductions (improvements) in ESS scores (Black & Hirshkowitz, 2005; Black et al., 2010; Dauvilliers et al., 2013, 2020; Harsh et al., 2006; Malhotra et al., 2020; Schweitzer et al., 2019; Szakacs et al., 2017; Thorpy et al., 2019; US Modafinil in Narcolepsy Multicenter Study Group, 2000). Despite this common utility, there are few studies that have evaluated the test–retest reliability of the ESS (Kendzerska et al., 2014).

The test–retest reliability of the ESS has primarily been investigated in healthy, community‐based samples (Ahmed et al., 2014; Johns, 1992; Knutson et al., 2006). A few studies (Campbell et al., 2018; Lee et al., 2020; Nguyen et al., 2006; Rozgonyi et al., 2021; Taylor et al., 2019; Walker et al., 2020) have evaluated the reliability of the ESS in sleep clinic patients with suspected sleep disorders by retrospective chart review with conflicting results. In real‐world clinic settings, multiple variables could change between assessments, such as the setting of the assessment (primary care setting versus sleep specialist setting), treatment interventions, caffeine use, and concomitant medications. Such factors could impact EDS and lead to greater variability in the ESS scores. These settings are not ideal for evaluating the test–retest reliability of the ESS in relation to its use as an outcome measure in a clinical trial setting. Therefore, it is necessary to examine the test–retest reliability of the ESS within a controlled clinical trial setting, in which multiple factors, such as those previously noted, would be uniform. However, there is a paucity of data ascertaining whether the ESS is reliable in a clinical trial setting and, furthermore, whether the reliability is observed in patients with sleep disorders other than suspected OSA.

Solriamfetol, a dopamine and noradrenaline re‐uptake inhibitor, is approved in the United States and European Union to improve wakefulness in adult patients with EDS associated with narcolepsy (75–150 mg/day) or OSA (37.5–150 mg/day) (Sunosi™ (solriamfetol) tablets Prescribing Information, 2019; Sunosi™ (solriamfetol) tablets Summary of Product Characteristics, 2020). In two randomised, placebo‐controlled, phase III trials and one long‐term open‐label extension (OLE) trial evaluating the effects of solriamfetol in participants with EDS associated with narcolepsy or OSA, ESS scores were included as a primary or co‐primary outcome measure (Malhotra et al., 2020; Schweitzer et al., 2019; Thorpy et al., 2019). The large sample sizes and structured nature of these studies provided an opportunity to assess the test–retest reliability of the ESS in a clinical sample in a clinical trial setting.

The aim of the present analysis was to examine the test–retest reliability of the ESS in participants with narcolepsy or OSA in a clinical trial setting, using the intraclass correlation coefficient (ICC) method (US Department of Health and Human Services, 2009). In addition, this analysis evaluated whether certain factors that might affect EDS, including treatment (with placebo or solriamfetol) and adherence to primary OSA therapy (i.e. adherence or non‐adherence), impact the reliability of the ESS.

## 2 METHODS

### 2.1 Study design

The present analysis includes data from phase III research from the solriamfetol clinical trial programme. This included two 12‐week, randomised, double‐blind, placebo‐controlled, phase III clinical trials: one in participants with narcolepsy (NCT02348593/EudraCT 2014‐005487‐15) (Thorpy et al., 2019); one in participants with OSA (NCT02348606/EudraCT 2014‐005514‐31) (Schweitzer et al., 2019), and one OLE trial in participants with narcolepsy or OSA (NCT02348632/EudraCT 2014‐005489‐31) (Malhotra et al., 2020).

All studies were approved by institutional review boards or ethics committees at each institution and were performed in accordance with the Declaration of Helsinki. All participants provided written informed consent. Complete descriptions of the study methods and primary results have been published previously (Malhotra et al., 2020; Schweitzer et al., 2019; Thorpy et al., 2019) and the methods are briefly summarised below.

### 2.2 Participants

For the 12‐week trials, eligible participants were adults (aged 18–75 years) diagnosed with narcolepsy (Type 1 or Type 2) or OSA and with ESS scores of ≥10. Additional key inclusion criteria included baseline mean sleep latency <25 min (narcolepsy) or <30 min (OSA) on the Maintenance of Wakefulness Test (MWT), and usual nightly total sleep time of ≥6 hr. Additional inclusion criteria were that participants with OSA were required to have current or history of prior (or attempted) use of a primary OSA therapy (i.e. to treat the underlying airway obstruction), including positive airway pressure, mandibular advance device, or surgical intervention. Participants without current primary OSA therapy use or a history of a successful surgical intervention to treat the underlying obstruction were required to have tried to use a primary OSA therapy for ≥1 month, with at least one documented adjustment to the therapy. At study entry, participants were instructed to maintain the same level of use of primary OSA therapy throughout the study. Key exclusion criteria included usual bedtime later than 1:00 a.m., night‐time or variable shift work, or any other clinically relevant medical, behavioural, or psychiatric disorder associated with EDS. Concomitant treatment with other medications that may affect the evaluation of EDS was not permitted.

For the OLE trial, participants with narcolepsy or OSA who had previously completed a phase II or phase III clinical trial of solriamfetol were eligible (Bogan et al., 2015; Ruoff et al., 2016; Schweitzer et al., 2019; Strollo et al., 2019; Thorpy et al., 2019). Due to differences in time between prior study completion and enrolment in the OLE trial, there were two groups. Group A enrolled in the OLE trial immediately after completion of one of the 12‐week phase III trials. Group B historically completed one of several other solriamfetol studies and was subsequently enrolled in the OLE trial.

### 2.3 Treatment

In the 12‐week trials, participants were randomised to receive placebo or solriamfetol 37.5 (OSA only), 75, 150, or 300 mg once daily for 12 weeks. In the OLE trial, solriamfetol treatment was initiated at 75 mg and titrated to 75, 150, or 300 mg during a 2‐week titration phase. The titration phase was followed by an open‐label maintenance phase (75, 150, or 300 mg), with a total study duration of 40 weeks (Group A) or 52 weeks (Group B).

### 2.4 ESS assessments

In all three trials, the ESS was administered at the investigative sites (sleep clinics) for all assessments. In the 12‐week trials, the ESS was assessed at baseline and at weeks 1, 4, 8, and 12. In the OLE trial, the ESS was assessed at baseline and at weeks 2, 14, 27, and 40 (Group A) or at weeks 2, 14, 26, 39, and 52 (Group B). Participants were instructed to complete the ESS based on the level of sleepiness they experienced over the past week (7‐day recall period) (Broderick et al., 2013; Plazzi et al., 2018).

### 2.5 Statistical analysis

For the 12‐week trials, data were analysed for the modified intent‐to‐treat (mITT) population, which was used for the primary efficacy analyses in these studies (Schweitzer et al., 2019; Thorpy et al., 2019) and was defined as participants who received one or more doses of study medication and had baseline and one or more postbaseline assessments. For the OLE trial, data were analysed for the safety population, defined as participants who received one or more doses of solriamfetol. The ICC estimates for ESS scores were calculated using postbaseline time‐point pairs. In the 12‐week trials, the time‐point pairs were weeks 4 and 8, weeks 8 and 12, and weeks 4 and 12. In the OLE trial, the time‐point pairs were weeks 14 and 26/27, weeks 26/27 and 39/40, and weeks 14 and 39/40 (week 52 was not included, as only Group B had data at this time‐point). All analyses included participants who had data at both visits for each time‐point pair.

The ICC estimates and 95% confidence intervals (CIs) were calculated for the overall population in each of the three trials and for the populations of the two 12‐week trials combined. For each 12‐week trial, the ICC estimates were also calculated by treatment (placebo or combined solriamfetol [all doses]). For participants with OSA (the full population of the 12‐week OSA trial and the OSA subgroup of the OLE trial), ICC estimates were calculated by adherence or non‐adherence to primary OSA therapy. Participants were categorised as adherent to primary OSA therapy if they had use of positive airway pressure therapy for ≥4 hr/night on ≥70% of nights, use of an oral appliance on ≥70% of nights, or receipt of an effective surgical intervention. Participants were categorised as non‐adherent if they had device use at a frequency/duration less than that described above, no use of a device at all, or a surgical intervention deemed no longer effective.

The ICC estimates and 95% CIs were calculated for each subsample using a two‐way mixed‐effects model, according to the method of Shrout and Fleiss (Shrout & Fleiss, 1979).

## 3 RESULTS

### 3.1 Participant population

In the 12‐week trials, the mITT populations included 231 participants with narcolepsy (placebo, *n* = 58; combined solriamfetol, *n* = 173) and 459 participants with OSA (placebo, *n* = 114; combined solriamfetol, *n* = 345). In the OLE trial, the safety population included 643 participants.

### 3.2 Participant demographics

Across all three trials, the majority of participants were White, not Hispanic or Latino, and primarily enrolled at sites in North America. In the 12‐week trial in participants with narcolepsy (mITT population), the majority of participants were female, mean age was ~36 years, and mean body mass index (BMI) was ~28 kg/m^2^ (Table 1). In the 12‐week trial of OSA (mITT population), the majority of participants were male, the mean age was ~54 years, and the mean BMI was ~33 kg/m^2^ (Table 1). In the OLE trial (safety population), 52% of participants were male, the mean age was ~49 years, and the mean BMI was ~32 kg/m^2^ (baseline data for the OLE trial have been previously reported (Malhotra et al., 2020)).

**TABLE 1:** Demographic and baseline clinical characteristics^a^

Characteristic	12‐week study – OSA		12‐week study – Narcolepsy		OLE
	Placebo (*n* = 114)	Combined solriamfetol (all doses) (*n* = 345)	Placebo (*n* = 58)	Combined solriamfetol (all doses) (*n* = 173)	Combined solriamfetol (all doses) (*n* = 643)^b^
Age, years, mean (*SD*)	54.0 (11.5)	53.8 (10.8)	36.2 (15.2)	36.2 (12.4)	49.3 (14.2)
Gender, *n* (%)					
Male	73 (64.0)	214 (62.0)	24 (41.4)	57 (32.9)	337 (52.4)
Female	41 (36.0)	131 (38.0)	34 (58.6)	116 (67.1)	306 (47.6)
BMI, kg/m^2^, mean (*SD*)	33.1 (5.3)	33.3 (5.3)	29.3 (5.8)	28.0 (5.8)	31.7 (5.9)
Race, *n* (%)					
American Indian or Alaska Native	1 (0.9)	0	0	2 (1.2)	n/a
Asian	4 (3.5)	13 (3.8)	0	6 (3.5)	15 (2.3)
Black or African American	26 (22.8)	61 (17.7)	10 (17.2)	23 (13.3)	109 (17.0)
Native Hawaiian or Other Pacific Islander	1 (0.9)	1 (0.3)	0	1 (0.6)	n/a
White	82 (71.9)	266 (77.1)	46 (79.3)	138 (79.8)	506 (78.7)
Multiple or Other	0	4 (1.2)	2 (3.4)	3 (1.7)	13 (2.0)
MWT sleep latency, min, mean (*SD*)^c^	12.6 (7.1)	12.5 (7.4)	6.2 (5.7)	8.0 (5.8)	n/a
ESS score, mean (*SD*)	15.6 (3.3)	15.1 (3.3)	17.3 (2.9)	17.2 (3.3)	15.9 (Group A) 15.9 (Group B)

BMI, body mass index; ESS, Epworth Sleepiness Scale; mITT, modified intent to treat; MWT, Maintenance of Wakefulness Test; n/a, not applicable; OLE, open‐label extension; OSA, obstructive sleep apnea; *SD*, standard deviation.

^a^ mITT population.

^b^ Data from Malhotra 2019.

^c^ For baseline mean sleep latency on MWT, OSA placebo, *n* = 111; OSA solriamfetol, *n* = 339; narcolepsy placebo, *n* = 57; narcolepsy solriamfetol, *n* = 170.

### 3.3 Test–retest reliability of ESS scores in the 12‐week and 1‐year trials (pooled data)

**FIGURE 1:**
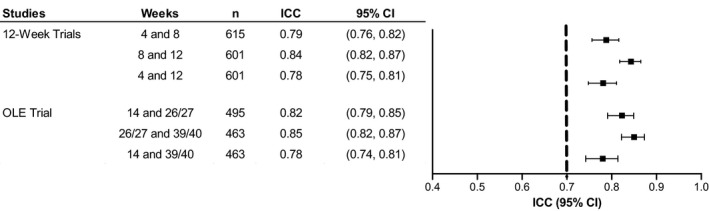
Test–retest reliability of ESS scores in solriamfetol 12‐week narcolepsy and OSA trials (pooled data) and long‐term OLE trial. Dashed line represents recommended threshold for acceptable test–retest reliability (ICC point estimates >0.7) (Terwee et al., 2007). CI, confidence interval; ESS, Epworth Sleepiness Scale; ICC, intraclass correlation coefficient; OLE, open‐label extension; OSA, obstructive sleep apnea

In the overall study populations, ICC estimates (95% CIs) ranged from 0.78 (0.75, 0.81) for weeks 4 and 12 to 0.84 (0.82, 0.87) for weeks 8 and 12 in the 12‐week trials (pooled data); and from 0.78 (0.74, 0.81) for weeks 14 and 39/40 to 0.85 (0.82, 0.87) for weeks 26/27 and 39/40 in the OLE trial (Figure 1).

### 3.4 Test–retest reliability of ESS scores in the 12‐week trials (by indication)

**FIGURE 2:**
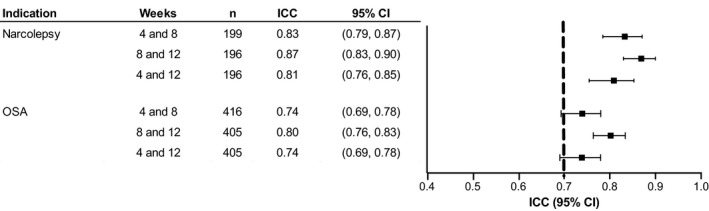
Test–retest reliability of ESS scores in solriamfetol 12‐week trials by indication (narcolepsy or OSA). Dashed line represents recommended threshold for acceptable test–retest reliability (ICC point estimates >0.7) (Terwee et al., 2007). CI, confidence interval; ESS, Epworth Sleepiness Scale; ICC, intraclass correlation coefficient; OSA, obstructive sleep apnea

In the individual 12‐week trials, the ICC estimates (95% CI) ranged from 0.81 (0.76, 0.85) for weeks 4 and 12 to 0.87 (0.83, 0.90) for weeks 8 and 12 for participants with narcolepsy and from 0.74 (0.69, 0.78) for weeks 4 and 8 to 0.80 (0.76, 0.83) for weeks 8 and 12 for participants with OSA (Figure 2).

### 3.5 Test–retest reliability of the ESS scores in the 12‐week trials (by indication and treatment)

**FIGURE 3:**
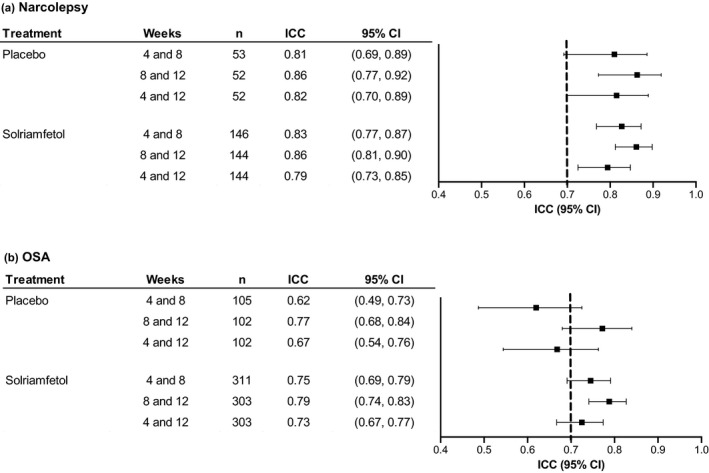
Test–retest reliability of ESS scores in solriamfetol 12‐week trials by indication (narcolepsy or OSA) and treatment (placebo or combined solriamfetol). Dashed line represents recommended threshold for acceptable test‐retest reliability (ICC point estimates >0.7) (Terwee et al., 2007). CI, confidence interval; ESS, Epworth Sleepiness Scale; ICC, intraclass correlation coefficient; OSA, obstructive sleep apnea

In the 12‐week trial in participants with narcolepsy, the ICC estimates (95% CI) ranged from 0.81 (0.69, 0.89) for weeks 4 and 8 to 0.86 (0.77, 0.92) for weeks 8 and 12 among participants who received placebo and from 0.79 (0.73, 0.85) for weeks 4 and 12 to 0.86 (0.81, 0.90) for weeks 8 and 12 among participants who received solriamfetol. In the 12‐week trial in participants with OSA, these values ranged from 0.62 (0.49, 0.73) for weeks 4 and 8 to 0.77 (0.68, 0.84) for weeks 8 and 12 among participants who received placebo and from 0.73 (0.67, 0.77) for weeks 4 and 12 to 0.79 (0.74, 0.83) for weeks 8 and 12 among participants who received solriamfetol (Figure 3).

### 3.6 Test–retest reliability of the ESS scores in the OSA populations in the 12‐week and 1‐year trials (by adherence/non‐adherence to primary OSA therapy)

**FIGURE 4:**
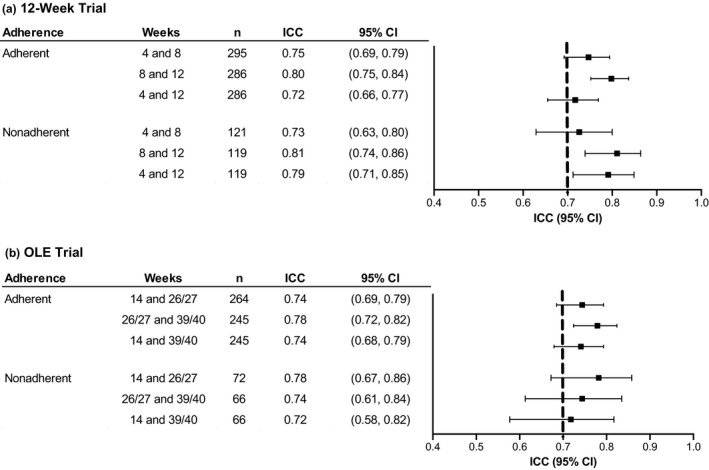
Test–retest reliability of ESS scores in solriamfetol 12‐week and long‐term OLE trials in participants with OSA by adherence/non‐adherence to primary OSA therapy. Dashed line represents recommended threshold for acceptable test–retest reliability (ICC point estimates >0.7) (Terwee et al., 2007). CI, confidence interval; ESS, Epworth Sleepiness Scale; ICC, intraclass correlation coefficient; OLE, open‐label extension; OSA, obstructive sleep apnea

In the 12‐week trial, the ICC estimates (95% CI) ranged from 0.72 (0.66, 0.77) for weeks 4 and 12 to 0.80 (0.75, 0.84) for weeks 8 and 12 among participants who were adherent and from 0.73 (0.63, 0.80) for weeks 4 and 8 to 0.81 (0.74, 0.86) for weeks 8 and 12 among participants who were non‐adherent to primary OSA therapy. In the OLE trial, the ICC estimates (95% CI) ranged from 0.74 (0.68, 0.79) for weeks 14 and 39/40 to 0.78 (0.72, 0.82) for weeks 26/27 and 39/40 among participants who were adherent and from 0.72 (0.58, 0.82) for weeks 14 and 39/40 to 0.78 (0.67, 0.86) for weeks 14 and 26/27 among participants who were non‐adherent (Figure 4).

## 4 DISCUSSION

In the present analysis, the ESS consistently demonstrated acceptable test–retest reliability across three large clinical trials of varying durations in clinical populations of participants with EDS associated with narcolepsy or OSA, regardless of treatment with placebo or a wake‐promoting agent (solriamfetol) and, in participants with OSA, independent of level of adherence or non‐adherence to primary OSA therapy. Specifically, the majority of ICC point estimates were >0.7, a threshold that has been recommended as a quality criterion for acceptable test–retest reliability (Terwee et al., 2007). The ICC point estimates below the threshold of <0.7 were observed only for placebo‐treated participants with OSA for weeks 4 and 8 (0.62) and weeks 4 and 12 (0.67); lower bound 95% CI values <0.7 were also observed in comparisons indicating reliability could potentially fall below this threshold at times. These findings support use of the ESS as a reliable measure of EDS in clinical trials.

In contrast with the present findings, some studies have reported different measures of variability in ESS scores among patients with diagnosed or suspected sleep disorders outside the clinical trial setting (e.g. in clinical practice) (Campbell et al., 2018; Lee et al., 2020; Nguyen et al., 2006; Rozgonyi et al., 2021; Taylor et al., 2019; Walker et al., 2020). Nguyen et al. (Nguyen et al., 2006) found that 41% of patients had an ESS score difference of ≥3 points between the first and second assessment, 23% had a difference ≥5, and 10% had a difference ≥7. Similarly, Campbell et al. (Campbell et al., 2018) found that 46%, 21%, and 8% of patients had differences ≥3, ≥5, and ≥7 points, respectively. Taylor et al. (Taylor et al., 2019) found that 46%–72% of patients had differences of ≥2 points and 3%–21% had differences of ≥8 points. Lee et al. (Lee et al., 2020) found that 56% of sleep clinic patients had a difference of ≥3 points; however, the ICC was 0.73 (95% CI 0.61, 0.82). Rozgonyi et al. (Rozgonyi et al., 2021) found that in patients referred to a sleep clinic (who may or may not have had a diagnosis of a sleep disorder), Lin’s concordance coefficient was 0.75 (scores of <0.9 indicate poor reliability) for pairs of ESS assessments that were an hour apart. Walker et al. (Walker et al., 2020) also found variability in individual ESS scores but found substantial agreement when the ESS was analysed in a binary fashion (i.e. sleepy or normal) using a ESS score cut‐off of ≥11; 89% of patients with ESS scores of ≥11 at the first assessment also had ESS scores of ≥11 at the second assessment (up to 90 days later). Several factors may account for the discrepancy in these findings. First, the methods for assessing test–retest reliability differed among studies. Notably, only one (Lee et al., 2020) used the ICC method, which was selected for use in the present study because it is an established statistical method for evaluation of test–retest reliability. Other methods, such as a naïve correlation analysis, are not sensitive to systematic differences in repeated measures. The present analysis evaluated data from prospective clinical trials, whereas most other studies retrospectively analysed data from chart reviews in clinical practice (Campbell et al., 2018; Lee et al., 2020; Nguyen et al., 2006; Taylor et al., 2019; Walker et al., 2020). Further, in the retrospective chart review analyses, there was no control of, or means of assessing, other factors that may have changed between the first and second assessments and impacted intra‐participant variability of EDS (e.g. change in total sleep time, medication, or caffeine use). Finally, there was variability in how the test was administered or completed. In many cases, the first and second ESS assessments were administered in different settings (primary care versus specialist) (Campbell et al., 2018; Lee et al., 2020; Nguyen et al., 2006; Taylor et al., 2019; Walker et al., 2020). Indeed, Taylor et al. (Taylor et al., 2019) found low ICC estimates (0.31–0.34) for assessments that were administered in different clinical settings; however, when both assessments occurred in the same setting, the ICC estimate was much higher at 0.82.

The present analysis also found that the ESS has acceptable test–retest reliability in participants with narcolepsy. This finding is consistent with a previous study that analysed data from a randomised, controlled clinical trial of pitolisant, modafinil, or placebo (van der Heide et al., 2015). Specifically, van der Heide et al. (van der Heide et al., 2015) found the ICC estimate for the ESS to be 0.83 among participants with narcolepsy. These consistent findings are likely attributable to the fact that both analyses were based on data from clinical trials, in which assessment settings were similar across all time‐points and factors that could affect variability of EDS (e.g. use of caffeine or other medications) were controlled.

A strength of the present analysis is the use of the ICC, an established statistical metric for evaluation of test–retest reliability. In addition, reliability was assessed in a large data set of participants with EDS‐associated sleep disorders across a variety of time intervals. Further, potential confounding factors that could contribute to intra‐participant variability, such as the setting in which the ESS was administered and medication/caffeine use, were controlled. Finally, the present analysis included an exploratory evaluation of test–retest reliability in subgroups defined by treatment status (placebo or a wake‐promoting agent) and adherence to primary OSA therapy. Despite these strengths, these clinical trials were not specifically designed to assess test–retest reliability of the ESS. Another limitation is that the generalisability of these findings to populations beyond patients with OSA or narcolepsy or to settings other than clinical trials is unknown.

In conclusion, the ESS consistently demonstrated acceptable test–retest reliability in clinical trial settings of OSA and narcolepsy across several months. Reliability remained acceptable across clinical trials of two different durations and in two different patient populations. Further, reliability was robust across subgroups defined by treatment (placebo or a wake‐promoting agent) and by adherence/non‐adherence to primary OSA therapy. These data from clinical trials suggest that the ESS is a reliable measure to assess the effects of treatment intervention on EDS in well‐conducted clinical trial settings.

## AUTHOR CONTRIBUTIONS

All authors contributed to the conception and design of the study. DM conducted the statistical analyses. All authors contributed to the interpretation of the results and critical revision of the manuscript for important intellectual content, and approved the final version of the manuscript.

## CONFLICT OF INTEREST

RR has received consultancy fees from Eisai; honoraria from Merck; and research funding from Jazz Pharmaceuticals, Merck, Actelion, Eisai, and Philips Respironics; and has served on the speakers' bureau for Merck and as a board member for Jazz Pharmaceuticals. KB, DM, and MB are employees of Jazz Pharmaceuticals who, in the course of their employment, have received stock options exercisable for, and other stock awards of, ordinary shares of Jazz Pharmaceuticals plc. DH and SM are former employees of Jazz Pharmaceuticals who, in the course of their employment, received stock options exercisable for, and other stock awards of, ordinary shares of Jazz Pharmaceuticals plc. JB is a part‐time employee of Jazz Pharmaceuticals and shareholder of Jazz Pharmaceuticals plc.

## Data Availability

All relevant data are provided with the manuscript and supporting files.
